# Immunogenicity of 60 novel latency-related antigens of *Mycobacterium tuberculosis*

**DOI:** 10.3389/fmicb.2014.00517

**Published:** 2014-10-08

**Authors:** Mᵃdel Mar Serra-Vidal, Irene Latorre, Kees L. C. M. Franken, Jéssica Díaz, Maria Luiza de Souza-Galvão, Irma Casas, José Maldonado, Cèlia Milà, Jordi Solsona, M. Ángeles Jimenez-Fuentes, Neus Altet, Alícia Lacoma, Juan Ruiz-Manzano, Vicente Ausina, Cristina Prat, Tom H. M. Ottenhoff, José Domínguez

**Affiliations:** ^1^Department of Microbiology, Institut d'Investigació en Ciències de la Salut Germans Trias i Pujol, Hospital Universitari Germans Trias i PujolBadalona, Spain; ^2^Department of Genetics and Microbiology, Universitat Autònoma de BarcelonaBellaterra, Spain; ^3^CIBER Enfermedades Respiratorias, Instituto de Salud Carlos IIIBadalona, Spain; ^4^Department of Immunohematology and Blood Transfusion/Department of Infectious Diseases, Leiden University Medical CenterLeiden, Netherlands; ^5^Unitat de Tuberculosi de Drassanes, Hospital Universitari Vall d'HebronBarcelona, Spain; ^6^Department of Preventive Medicine, Institut d'Investigació en Ciències de la Salut Germans Trias i Pujol, Hospital Universitari Germans Trias i PujolBadalona, Spain; ^7^Serveis Clínics de BarcelonaBarcelona, Spain; ^8^Department of Pneumology, Institut d'Investigació en Ciències de la Salut Germans Trias i Pujol, Hospital Universitari Germans Trias i PujolBadalona, Spain

**Keywords:** tuberculosis, latent tuberculosis infection, immune response, antigenic stimulation, interferon-γ

## Abstract

The aim of our work here was to evaluate the immunogenicity of 60 mycobacterial antigens, some of which have not been previously assessed, notably a novel series of *in vivo*-expressed *Mycobacterium tuberculosis* (IVE-TB) antigens. We enrolled 505 subjects and separated them in individuals with and without latent tuberculosis infection (LTBI) vs. patients with active tuberculosis (TB). Following an overnight and 7 days stimulation of whole blood with purified recombinant *M. tuberculosis* antigens, interferon-γ (IFN-γ) levels were determined by ELISA. Several antigens could statistically significantly differentiate the groups of individuals. We obtained promising antigens from all studied antigen groups [dormancy survival regulon (DosR regulon) encoded antigens; resuscitation-promoting factors (Rpf) antigens; IVE-TB antigens; reactivation associated antigens]. Rv1733, which is a probable conserved transmembrane protein encoded in DosR regulon, turned out to be very immunogenic and able to discriminate between the three defined TB status, thus considered a candidate biomarker. Rv2389 and Rv2435n, belonging to Rpf family and IVE-TB group of antigens, respectively, also stood out as LTBI biomarkers. Although more studies are needed to support our findings, the combined use of these antigens would be an interesting approach to TB immunodiagnosis candidates.

## Introduction

Tuberculosis (TB) remains one of the most death-causing microorganism worldwide (World Health Organization, 2013). The increasing numbers of drug-resistant TB cases evidence that there is an urgent need for effective diagnosis, drugs and vaccines (Mwaba et al., 2011; Abubakar et al., 2013). The control of latent TB, a stage in which a person is infected with *Mycobacterium tuberculosis* (*Mtb*) but does not currently have active disease, plays an important role for disease control, since dormant bacilli are an enormous reservoir of potential TB cases (Rustad et al., 2009).

*M.tb* can live in a latent stage without causing any clinical symptom and has a potential of reactivation during all the infected individual lifetime. In fact, about one third of the world population is considered to be latently infected (Corbett et al., 2003). The diagnosis of latent tuberculosis infection (LTBI) through the classic tuberculin skin test (TST) has a lack of specificity, and its sensitivity is low in high-risk groups of progression to active TB. The new interferon (IFN)-γ release assays (IGRAs) are immunodiagnostic methods based on the *in vitro* quantification of the cellular immune response. The detection of IFN-γ released by sensitized T cells stimulated with specific *M.tb* antigens enables the identification of infected individuals. The main antigens used in IGRAs, the 6-kDa *M.tb* early-secreted antigenic target (ESAT)-6 protein, 10-kDa culture filtrate protein (CFP-10), coded in the region of difference (RD) 1, and TB7.7, coded in RD11, are present in *M.tb* but not in any *Mycobacterium bovis* bacillus Calmette-Guérin (BCG) vaccine strain nor in the majority of non-tuberculous mycobacteria (Andersen et al., 2000). Although their specificity is better than in TST, IGRAs do not discriminate between active disease and LTBI (Latorre et al., 2009) and do not clearly distinguish between a recently acquired infection and remote LTBI (Esmail et al., 2012; Pollock et al., 2013). Moreover, their sensitivity barely exceed 80%, and the response level against the antigens used does not seem to indicate high risk of progression to active TB. There is a need of new TB antigens as biomarkers for LTBI immunodiagnosis.

During LTBI, *M.tb* is contained within granulomas, which are formed by activated macrophages and other host components that isolate the infected cells in an organized structure and create an environment that suppresses *M.tb* replication (Esmail et al., 2012). Bacilli must adapt to a variety of environment stresses including reduced oxygen tension, iron limitation, nutrient deprivation, low pH and production of host factors such as nitric oxide and carbon monoxide. Some *in vitro* models demonstrated that *M.tb* is capable of an extensive repertoire of metabolic realignments to enter a defined non-replicating state. The initial response of *M.tb* is encoded by the dormancy survival regulon (DosR, also called DevR, Rv3133c), which leads to induction of a set of ~50 genes, many of unknown function. DosR controls the expression of genes that allow the bacteria to use alternative energy sources, especially lipids, and genes encoding factors that are selectively recognized by T cells from humans with LTBI (Ernst, 2012). This initial response is followed by a more extensible and more stable response called Enduring Hypoxic Response (EHR), which is comprised of 230 genes involved in the control of the regulatory factors and enzymatic machines of the long-term bacteriostasis program of non-replicating *M.tb* (Rustad et al., 2008). The antigens expressed by *M.tb* vary during the continued pressure mounted by host immune response in the course of the infection (Honer zu Bentrup and Russell, 2001; Demissie et al., 2006). Using *in vitro* models which mimic the conditions that the tubercle bacillus encounter within the host as infection progresses from latency to active disease, some infection phase-dependent genes have been identified and believed to be candidates for immunodiagnostics or for future vaccines (Mukamolova et al., 1998; Zvi et al., 2008; Ottenhoff and Kaufmann, 2012).

DosR regulon is crucial for rapid resumption of growth by involving resuscitation-promoting factors (Rpf) once *M.tb* exits the hypoxic non-respiring state. *M.tb* contains five Rpf -like proteins that are implicated in resuscitation of this microorganism from dormancy to reactivation via a mechanism involving hydrolysis of the peptidoglycan by Rpfs and partnering proteins (Ernst, 2012).

The *in vitro* models mentioned above supposed to recapitulate relevant environmental stress conditions that *M.tb* encounters upon host infection, which allow to identify differentially regulated *M.tb* genes. However, they present some limitations: many of these environmental stress factors may not be well known yet; there may be additive or synergistic effects between multiple stress factors *in vivo* that may easily be missed when studied in isolation *in vitro*; and certain key features of host response-induced stress cannot readily be recapitulated *in vitro*, including granuloma formation and TB necrosis (Commandeur et al., 2013).

For these reasons, different approaches have been developed to analyze the gene expression profiles of intracellular *M.tb* using infected human or murine macrophages, infected murine tissue or artificial granuloma mouse models (Schnappinger et al., 2003; Karakousis et al., 2004; Talaat et al., 2004; Cappelli et al., 2006). Specifically, several *M.tb* genes have been found to be differentially expressed in the lungs of mice strains with high susceptibility to TB during *in vivo* infection, the so-called *in vivo-expressed M.tb* (IVE-TB) genes. Interestingly, some of these IVE-TB genes had been also described as induced for nutrient deprivation in *in vitro* models (Commandeur et al., 2013).

Some *M.tb* infection phase-dependent antigens have already been tested in whole-blood assays or in peripheral blood mononuclear cells and may be differentially recognized in individuals with different TB status, that is, subjects with no risk of *M.tb* infection, LTBI individuals and active TB patients (Leyten et al., 2006; Lin et al., 2007, 2009; Black et al., 2009; Schuck et al., 2009; Goletti et al., 2010; Commandeur et al., 2013). The response is commonly measured through the release of IFN-γ. Additionally, IFN-γ plays a central role in the protection against *M.tb* (Cooper et al., 1993; Flynn et al., 1993; Kaufmann, 2001).

However the studies conducted *in vivo*, mice models of latency, seem to merely recapitulate primary disease and are closer to human HIV-TB co-infection. The infectious forms of TB arise after an adequate immune response, which itself may contribute to tissue destruction and cavitation. Hence, there has been a move away from considering IFN-γ as protective, except in the first encounter with the tubercle bacillus, with a renewed emphasis on polyfunctional T cells to contain TB infection (Ernst, 2012; Kaufmann, 2012). However, the ease of measuring IFN-γ gives it applicability to diagnostic tests.

The study of the immune response to the potential immunogenic *M.tb* antigens described above will enlarge our knowledge and will get us closer to the validation of a diagnostic LTBI candidate antigen. We hypothetized that these antigens expressed in latency conditions and involved in reactivation of the dormant bacterial will mainly induce IFN-γ response in LTBI infected patients, and not in no LTBI individuals.

This prospective study aims to evaluate the whole blood IFN-γ response to 60 *M.tb* recombinant antigens, the immunogenicity of some of them has not yet been assessed: 6 DosR regulon-encoded antigens, 12 TB reactivation-associated antigens, 1 Rpf antigen, 1 starvation antigen, 6 other stress response-associated TB antigens and 34 IVE-TB antigens (2 of those were EHR and 3 were EHR/starvation), in order to identify potential candidates for new LTBI diagnostic methods. We enrolled subjects with LTBI, active TB patients and controls not *M.tb* infected.

## Materials and methods

### Study population

We prospectively recruited 578 patients from contact-tracing studies, LTBI screening (such as immigrants from endemic areas or health-care workers) and active TB patients between October 2010 and May 2013. A detailed questionnaire from each subject was collected, including age, birth country, previous TST, BCG vaccination status, history of prior active TB, chest radiography, and other medical conditions (Table 1). A total of 8 mL of whole blood was collected in a heparinized tube from each participant. The study obtained approval of the Hospital Universitari Germans Trias i Pujol Ethics Committees (Ref. 10/00214-28/03/2010). They supervised that all the experiments were performed according to the regulatory standards. All the study participants gave written informed consent before entering the study.

**Table 1:** **Demographic characteristics and clinical details for individuals in this study**.

	**No TB infected individuals (*n* = 97)**	**LTBI infected (*n* = 306)**	**Active TB patients (*n* = 102)**
**GENDER**			
Male	61 (62.9)	131 (42.8)	73 (71.6)
Female	36 (37.1)	175 (57.2)	29 (28.4)
**ORIGIN**			
Spain	31 (32)	171 (55.9)	43 (42.2)
Africa	2 (2.1)	15 (4.9)	15 (14.7)
America	16 (16.5)	40 (13.1)	13 (12.7)
South-East Asia	15 (15.5)	16 (5.2)	5 (4.9)
Europe	2 (2.1)	11 (3.6)	0 (0)
Eastern Mediterranean	21 (21.6)	41 (13.4)	23 (22.5)
Western pacific	10 (10.3)	12 (3.9)	3 (2.9)
**BCG VACCINATION**			
Yes	71 (73.2)	138 (45.1)	36 (35.3)
No	25 (25.8)	167 (54.6)	57 (55.9)
Unknown	1 (1.0)	1 (0.3)	9 (8.8)
**QFN RESULT**			
Positive	0 (0)	182 (59.5)	21 (20.6)
Negative	97 (100)	123 (40.2)	5 (4.9)
Indeterminate	0 (0)	0 (0)	0 (0)
Not done	0 (0)	1 (0.3)	76 (74.5)
**PREVIOUS TB**			
Yes	0 (0)	1 (0.3)	11 (10.8)
No	96 (99)	238 (77.8)	91 (89.2)
Unknown	1 (1.0)	67 (21.9)	0 (0)
**IMMUNOSUPPRESSION**			
No	94 (96.9)	300 (98)	94 (92.2)
HIV+	1 (1.0)	2 (0.7)	2 (2.0)
Other	2 (2.1)	4 (1.3)	6 (5.9)
**TB CLINICAL FORM**			
Pulmonar	0 (0)	0 (0)	84 (82.4)
Ganglionar	0 (0)	0 (0)	7 (6.9)
Pleural	0 (0)	0 (0)	1 (1.1)
Pulmonar and ganglionar	0 (0)	0 (0)	2 (2.0)
Pulmonar and pleural	0 (0)	0 (0)	2 (2.0)
Disseminated	0 (0)	0 (0)	5 (4.9)
Erythema nodosum	0 (0)	0 (0)	1 (1.0)

*Number of individuals and percentage (%) are indicated*.

Participants were classified, following Spanish Society of Respiratory Pathology (SEPAR) guidelines (Ruiz-Manzano et al., 2008) and also Centers for Disease Control and Prevention (CDC) recommendations (Centers for Disease Control and Prevention, 2000), in four groups depending on the TB status, as is described in detail below. The method used for diagnosing LTBI was TST (PPD RT23, Statens Serum Institute, Copenhagen, Denmark) and QuantiFERON-TB Gold *In Tube* (QFN; QIAGEN, Düsseldorf, Germany).

The following individuals were included as no LTBI: (a) individuals from LTBI screening studies, who tested QFN negative and TST under 10 or 15 mm (depending on the absence or presence of BCG vaccination, respectively); and (b) individuals who reported contact with a TB patient and with negative QFN, whose TST was under 5 mm. All patients included were HIV negative.

As LTBI were included: (a) individuals who reported a contact with a TB patient or from LTBI screening studies, who tested QFN positive; (b) individuals who reported an intense contact with a TB patient, with negative QFN and TST higher than 5 mm (if the subject is BCG vaccinated, the index case has to be smear-positive; if it is smear-negative, TST has to be higher than 15 mm); (c) individuals from LTBI screening studies, who tested QFN negative and whose TST converted (by definition, from under 10 mm to above 10 mm with a change of 6 mm); and (d) individuals from LTBI screening studies, who tested QFN negative and TST positive (higher than 10 mm in non BCG-vaccinated and recent immigrants; and higher than 15 mm in BCG-vaccinated).

Individuals with pulmonary or extrapulmonary active TB, clinically, radiologically and/or microbiologically diagnosed (World Health Organization, 2013) were included.

### *Mycobacterium tuberculosis* antigens

A total of 60 *M.tb* recombinant latency-related antigens were evaluated (Table 2): 6 DosR regulon-encoded antigens, 12 TB reactivation-associated antigens, 1 Rpf antigen, 1 starvation antigen, 6 other stress response-associated TB antigens and 34 IVE-TB antigens (two of them were EHR and three were EHR/starvation). They were previously produced at the Department of Infectious Diseases, Leiden University Medical Center following the methododology previously described (Franken et al., 2000). Briefly, antigens were selected from RNA microarray studies after inducing hypoxic conditions in a *M.tb* liquid culture. The selected genes were cloned in *Escherchia coli* and antigens were overexpressed and purified by immobilized metal chelate affinity chromatography. Some antigens were prepared as two or three recombinant protein fragments owing to their large sizes (C, middle [M], and N termini). For IVE-TB genes, mice were infected with *M.tb* and RNA was isolated from mouse lung tissue (Commandeur et al., 2013). After a RT-PCR, highly or differentially expressed genes were selected and cloned by Gateway technology (Invitrogen, Carlsbad, US) in *E. coli* and antigens were obtained as explained before.

**Table 2:** **Description of the 4 control and 60 *M.tb* recombinant antigens tested, included DosR regulon-encoded (*n* = 6), TB reactivation-associated (*n* = 12), Rpf (*n* = 1), starvation (*n* = 1), other stress response-associated (*n* = 6), and IVE-TB antigens (*n* = 34) (Function information source: http://www.ncbi.nlm.nih.gov/nuccore)**.

**Antigen name**	**Function**
**CONTROL ANTIGENS (*n* = 4)**	
PPD	Purified protein derivative
Rv0288 (TB10.4)	Low molecular weight protein antigen belongs to the ESAT-6 (esx) family
Rv3875/3874 (ESAT-6/CFP-10)	6-kDa early secretory antigenic target/10 kDa culture filtrate (fusion protein)
Rv3804c (Ag85A)	Secreted antigen. Fibronectin binding protein acyltransferase activity
***M.tb* RECOMBINANT ANTIGENS (*n* = 60)**	
**DosR**	
*Rv0570c*	Probable ribonucleoside-diphosphate reductase C-ter (aa 333-692)
*Rv0570n*	Probable ribonucleoside-diphosphate reductase N-ter (aa 1-354)
**Rv1733**	Probable conserved transmembrane protein
*Rv2626*	Conserved hypothetical protein
*Rv2627*	Conserved hypothetical protein
*Rv2628*	Hypothetical protein
**Reactivation**	
*Rv0140*	Conserved hypothetical protein
*Rv0251*	Possible heat shock protein
*Rv0384*	Heat shock protein F84.1
*Rv0753*	Methylmalmonate semialdehyde dehydrogenase
**Rv1471**	Thioredoxin reductase
*Rv1874*	Hypothetical protein
*Rv1875*	Conserved hypothetical protein
Rv2465^*^	Phosphopentose isomerase
*Rv2466*	Conserved hypothetical protein
**Rv2662**	Hypothetical protein
*Rv3223*	ECF subfamily sigma subuint
**Rv3862**	Possible transcriptional regulatory protein WHIB6
**Rpf**	
**Rv2389**	Possible resuscitation promoting factor D
**Starvation**	
**Rv2660**	Hypothetical protein
**Other *M.tb* stress induced**	
**Rv0244**	Probable Acyl-coA dehydrogenase
*Rv0767*	Conserved hypothetical protein
**Rv1909**	Ferric uptake regulation protein
*Rv2745*	Possible transcriptional regulatory protein
**Rv2913**	Possible D-amino acid amonohydrolase
*Rv3406*	Probable dioxygenase
**IVE-TB**	
**Rv0847**	Probable LpqS, lipoprotein
**Rv0967**	Copper-sensitive operon repressor
*Rv0990*	Hypothetical protein
*Rv0991*	Conserved serine rich protein
*Rv1170*	N-acetyl-1-D-myo-inosityl-2-amino-2-deoxy-alpha-D-glucopyranoside deacetylase MshB
Rv1284^*^^≠^	Conserved hypothetical protein
*Rv1363*	Possible membrane protein
*Rv1403*	Putative methyltransferase
**Rv1806**	PE family protein PE20
*Rv1955*	Possible toxine HigB
Rv1956^*^^≠^	Possible antitoxin HigA
*Rv1957*	Hypothetical protein
Rv2034^*^^≠^	ArsR repressor protein
*Rv2035*	Conserved hypothetical protein
*Rv2225*	3-methyl-2-oxobutanoate hydroxymethyltransferase (panB)
Rv2324^*^	Probable transcriptional regulatory protein (probably AsnC-family)
*Rv2380c*	Peptide synthetase mbtE C-ter (aa 1120-1682)
**Rv2380M**	Peptide synthetase mbtE middle part (aa 560-1140)
*Rv2380N*	Peptide synthetase mbtE N-ter (aa 1-580)
*Rv2435c*	Probable cyclase (adenylate or guanylate cyclase) C-ter (aa 340-730)
**Rv2435n**	Probable cyclase (adenylate or guanylate cyclase) N-ter (aa 1-360)
*Rv2558*	Conserved protein
**Rv2642**	Possible transcriptional regulatory protein
*Rv2643*	Probable arsenic-transport integral membrane protein ArsC
*Rv2658*	Possible prophage protein
*Rv2737c*	Recombination protein recombinase A (recA) C-ter (aa 400-790)
*Rv2737n*	Recombination protein recombinase A (recA) N-ter (aa 1-420)
*Rv2838*	Probable ribosome-binding factor A (P15B protein)
*Rv2982*	Probable glycerol-3-phosphate dehydrogenase (gpdA2)
*Rv3353*	Conserved hypothetical protein
*Rv3420*	Ribosomal-protein-alanine acetyltransferase rimI
Rv3515^*^	Fatty-acid-CoA synthase
*Rv3536*	Probable hidratase
*Rv717*	30S ribosomal protein S14 RpsN1

*M.tb recombinant antigens with Rv designation in bold induced a relevant IFN-γ response in this study*.

* *also EHR*.

≠ also starvation

Apart from those *M.tb* recombinant antigens, we used 4 control antigens for which immunogenicity and specificity to *M.tb* is well defined: the fusion protein ESAT-6 [Rv3875]/CFP-10[Rv3874], Ag85A[Rv3804c], TB10.4[Rv0288], and PPD (PPD RT 23, Serum Institute, Copenhagen, Denmark).

Antigens were reconstituted in sterile phosphate buffered saline, to a concentration of 50 μg/mL and stored at −20°C. The 60 latency-related antigens were randomly grouped into 10 batches of 6, and the individuals tested randomly selected. Thus, the whole blood from each patient was stimulated with six antigens, and the four control antigens as well.

### Whole blood assay

400 μL of whole blood were transferred to a 48 well culture plate (Nunc, St. Louis, US) and control antigens were added at a final concentration of 10 μg/mL except for PPD, that was added at 1 μg/mL. *M.tb* recombinant antigens were tested at a final concentration of 10 μg/mL. A negative (RPMI medium; PAA, Pasching, Austria) and a positive control of immunity (phytohemagglutinn, Invitrogen, Carlsbad, US) were included. This procedure was performed in two different plates: one plate was incubated in a 5% CO_2_ incubator a 37°C overnight (18 h, short-term stimulation) and the other for 7 days (long-term stimulation). In the long-term incubation plate, blood was previously diluted 1:5 with RPMI 1640 medium supplemented with L-glutamine, penicillin and streptomycin (Weir et al., 2003). After incubation time, supernatants were then collected and stored at −80°C until tested.

### Determination of IFN- γ by ELISA

The measurement of the amount of IFN-γ released following the antigenic stimulation was evaluated by the commercial ELISA included in the QFN kit and data are presented as pg/mL after subtraction of the negative control. We considered a valid result when the value of the negative control was under 50 pg/ml. The cut-off value for high level of IFN-γ response was arbitrarily set at 20 pg/ml, taking as reference the QFN cut-off.

### Statistical analysis

The production level of IFN-γ was compared between the groups included in the study. Median and range of the cytokine production was calculated and Mann Withney test was used for pair-wise comparisons and Kruskall Wallis test was used for multiple comparisons. A *P*-value <0.05 was considered significant. Data were analyzed using SPSS statistical software (IBM SPSS Statistics 20; IBM Corporation, NY, USA). Graphical representation is based on GraphPad Prism version 4 (GraphPad Software, Inc., San Diego, CA).

## Results

From the 578 participants, 60 were not tested for the determination of IFN-γ because of insufficient samples, eight subjects did not fulfill the inclusion criteria and five subjects were excluded from the study because the amount of IFN-γ in the negative control was too high. In the patients PHA induced high responses, which ratifies the validity of our methodology.

### Immunogenicity of control TB antigens

We included four control antigens in our study. Significant differences in the IFN-γ responses elicited by all of them could be observed between the three study groups after short-term and after long-term stimulation (Tables 3, 4). The antigens that elicited a higher response were PPD and the fusion protein ESAT-6/CFP-10, followed by TB10.4 and Ag85A.

**Table 3:** **Median levels of IFN-γ (pg/ml), minimum and maximum values (in brackets) elicited in no TB infected individuals, subjects with LTBI and active TB patients by the antigens, when tested after short-term stimulation**.

**Antigen**	**No TB infection**		**TB infection**		**Active TB**		***p*-value**
	***n***	**Median**	***n***	**Median**	***n***	**Median**	
**CONTROL**							
PHA	95	907.0 (26.3, 5396.5)	294	807.5 (19.5, 12132.0)	98	139.3 (0.0, 2943.5)	0.000
PPD	90	25.5 (0.0, 502.5)	275	57.3 (0.0, 1299.0)	95	19.5 (0.0, 1198.0)	0.000
TB10.4	94	3.5 (0.0, 125.5)	288	9.3 (0.0, 581.5)	91	3.0 (0.0, 355.7)	0.003
ESAT6/CFP10	94	6.5 (0.0, 591.0)	280	20.3 (0.0, 2758.5)	91	5.5 (0.0, 752.0)	0.005
Ag85A	88	0.0 (0.0, 69.5)	262	1.0 (0.0, 145.0)	78	0.5 (0.0, 311.0)	0.011
**DosR**							
Rv2627	16	0.8 (0.0, 5.0)	54	1.0 (0.0, 34.5)	6	0.0 (0.0. 0.5)	0.044
Rv1733	4	32.8 (12.0, 66.5)	20	69.8 (0.5, 733.0)	9	3.0 (0.0, 32.5)	0.001
**REACTIVATION**							
Rv1471	4	3.8 (0.0, 9.0)	19	1.5 (0.0, 19.0)	8	0.0 (0.0, 0.5)	0.010
Rv1874	4	2.3 (0.0, 3.0)	19	1.0 (0.0, 9.5)	7	0.0 (0.0, 0.5)	0.009
Rv3862	7	2.0 (0.0, 14.0)	21	4.5 (0.0, 32.5)	9	0.0 (3.0, 4.0)	0.019
**IVE-TB**							
Rv0967	8	1.8 (0.0, 13.0)	19	3.0 (0.0, 253.0)	10	0.0 (0.0, 1.5)	0.017
Rv1806	7	5.0 (0.0, 35.5)	15	7.5 (0.0, 56.5)	7	1.0 (0.0, 3.5)	0.023
Rv1957	4	2.8 (0.0, 5.5)	19	0.5 (0.0, 18.5)	8	0.0 (0.0, 0.5)	0.020

*N indicates the number of subjects in each group. Data were analyzed by Kruskal Wallis test for the comparison of no TB infection, TB infection, and active TB groups of patients. Only significant differences (p < 0.05) were included*.

**Table 4:** **Median levels of IFN-γ (pg/ml), minimum and maximum values (in brackets) elicited in no TB infected individuals, subjects with LTBI and active TB patients by the antigens, when tested after long-term stimulation**.

**Antigen**	**No TB infection**		**TB Infection**		**Active TB**		***p*-value**
	***n***	**Median**	***n***	**Median**	***n***	**Median**	
**CONTROL**							
PHA	94	1136.0 (189.8, 6443.0)	293	1013.9 (296.5, 12135.5)	102	854.3 (0.0, 4026.3)	0.000
PPD	89	13.5 (0.0, 925.5)	275	59.5 (0.0, 2246.0)	99	60.0 (0.0, 1681.2)	0.000
TB10.4	94	1.6 (0.0, 115.0)	288	3.2 (0.0, 1154.0)	100	1.5 (0.0, 675.0)	0.004
ESAT6/CFP10	93	86.0 (0.0, 2818.5)	280	163.5 (0.0, 6440.0)	100	29.7 (0.0, 3966.0)	0.000
**DosR**							
Rv0570c	8	0.0 (0.0, 4.5)	19	0.0 (0.0, 5.0)	5	1.5 (0.5, 5.5)	0.012
Rv1733	4	345.3 (178.5, 2766.5)	20	356.5 (3.5, 2150.0)	10	7.3 (1.0, 394.5)	0.010
**REACTIVATION**							
Rv1471	3	2.0 (1.5, 38.5)	15	1.0 (0.0, 25.5)	8	10 (0.0, 1.5)	0.028
**Rpf, OTHER STRESS-INDUCED**							
Rv0244	8	17.8 (5.0, 50.0)	24	7.3 (0.0, 63.5)	8	0.3 (0.0, 4.5)	0.005
Rv2389	7	148.5 (12.0, 257.0)	16	135.8 (1.5, 610.5)	5	22.5 (5.0, 59.5)	0.046
**IVE-TB**							
Rv0847	7	6.5 (2.5, 182.5)	21	3.5 (0.0, 298.5)	9	1.0 (0.0, 111.0)	0.011
Rv2558	4	1.0 (0.0, 2.5)	17	0.0 (0.0, 2.0)	9	0.5 (0.0, 6.5)	0.038
Rv2642	3	23.5 (16.5, 315.0)	15	33.5 (1.5, 191.0)	8	1.0 (0.0, 3.5)	0.002

*N indicates the number of subjects in each group. Data were analyzed by Kruskal Wallis test for the comparison of no TB infection, TB infection, and active TB groups of patients. Only significant differences (p < 0.05) were included*.

### Immunogenicity of DosR regulon-encoded antigens

**Figure 1:**
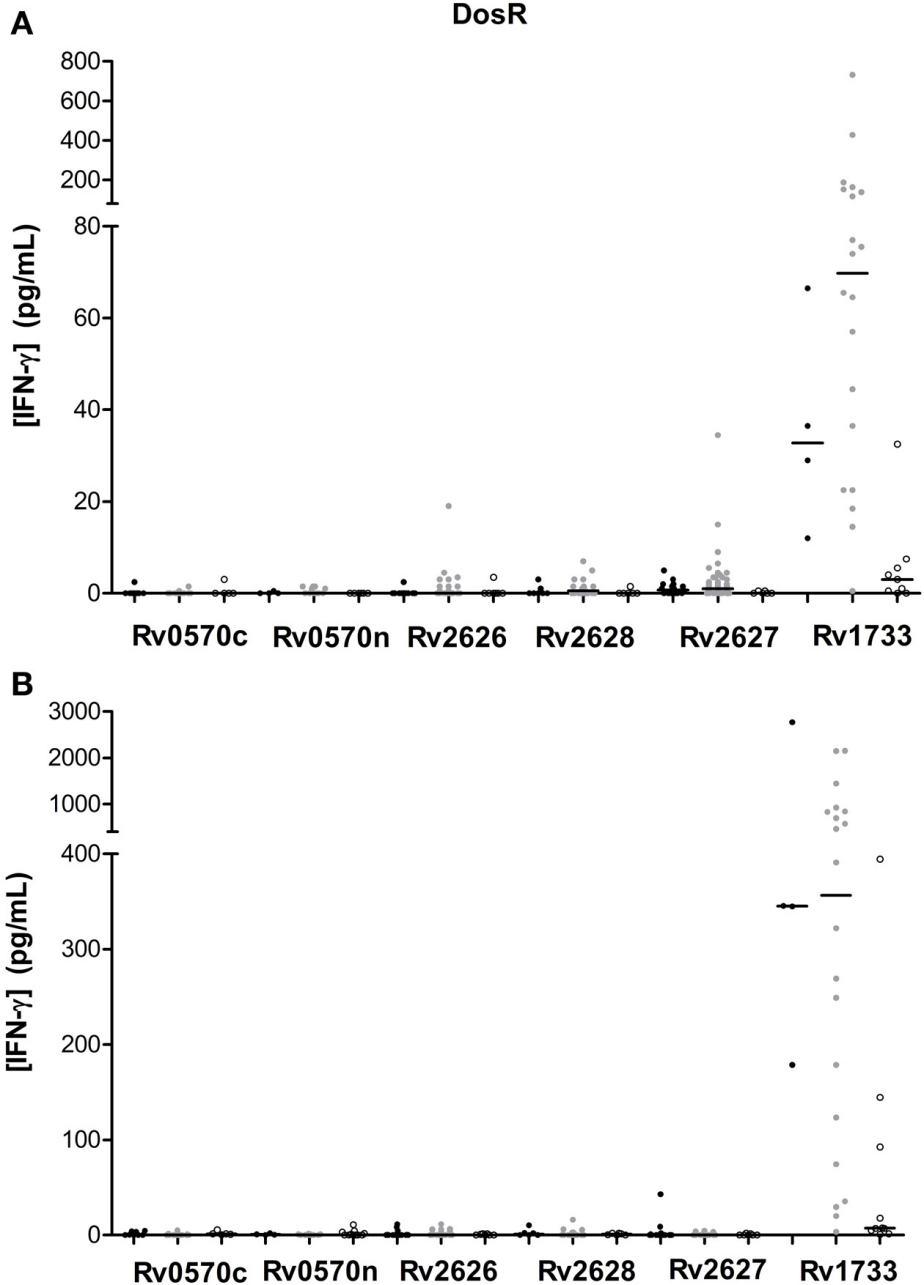
**DosR regulon-encoded antigen stimulated IFN-γ response (pg/ml) after short (A) or long-term (B) incubation of whole blood from individuals without LTBI (black dots), with LTBI (gray dots), and active TB patients (empty dots)**. The horizontal line represents the median.

We evaluated six different DosR regulon-encoded antigens (Figure 1). Two of them were the C-ter and N-ter domain of a latency antigen (Table 2). There were three antigens which presented a differentiated response depending on the TB status group: Rv1733, Rv2627, and Rv0570c (Tables 3, 4). While Rv1733 discriminated between groups when the stimulation was either short-term or long-term, the discrimination of Rv2627 only was significant after short-term stimulation, and Rv0570c was only after long-term stimulation (although with a low IFN-γ response). The best discriminatory response was elicited when whole blood was stimulated overnight with Rv1733 (*p* = 0.001), where the infected individuals response was clearly much higher than in TB patients and higher than the response produced by non-infected individuals. When incubated for 7 days, Rv1733 also induced a high amount of IFN-γ released in infected individuals, being the response much higher than in TB patients. Rv1733 turned out to be a strong immunoresponse inducer, a promising LTBI biomarker and a promising antigen in discriminating between LTBI individuals, active TB patients and non-infected subjects. The Rv1733, considering the 20 pg/ml as a cut-off, accurately predicted 85% (17/20) of LTBI patients in short-term stimulation; and 95% (19/20) in long-term stimulation.

### Immunogenicity of TB reactivation-associated antigens

**Figure 2:**
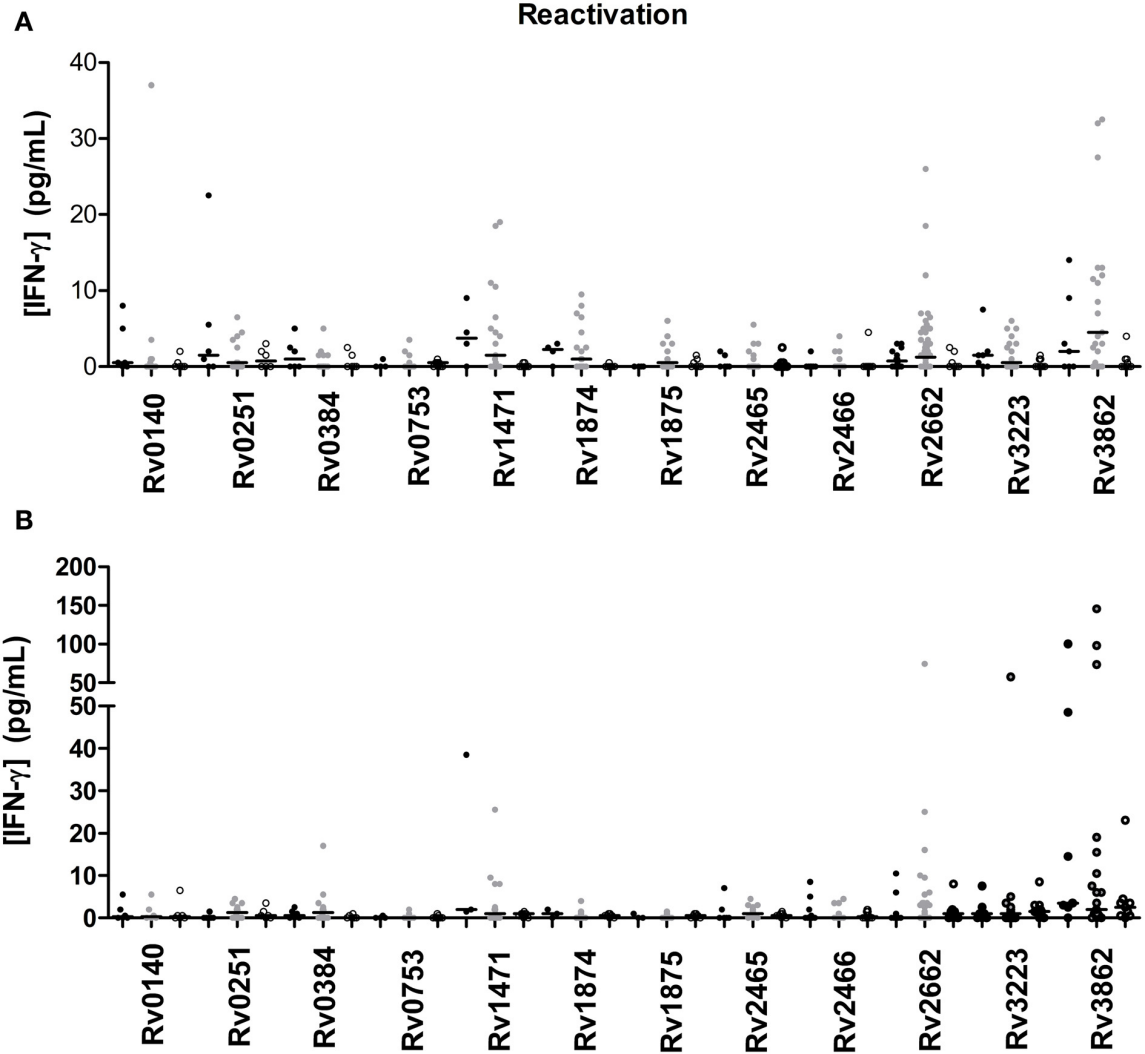
**TB reactivation-associated antigen stimulated IFN-γ response (pg/ml) after short (A) or long-term (B) incubation of whole blood from individuals without LTBI (black dots), with LTBI (gray dots), and active TB patients (empty dots)**. The horizontal line represents the median.

A total of 12 TB reactivation-associated antigens were evaluated in the study (Figure 2). In general, recognition of these antigens was poor in subjects with LTBI and TB patients. However, there were several antigens which showed different IFN-γ production depending on the group of individuals (Tables 3, 4). While Rv1471, Rv1874, Rv1875, Rv2662, and Rv3862 induced differentiate response in infected individuals in short-term stimulation; Rv1471, Rv2622, and Rv3862 induced differentiate and high response in infected individuals in long-term stimulation.

### Immunogenicity of Rpf, starvation and other stress response-associated antigens

**Figure 3:**
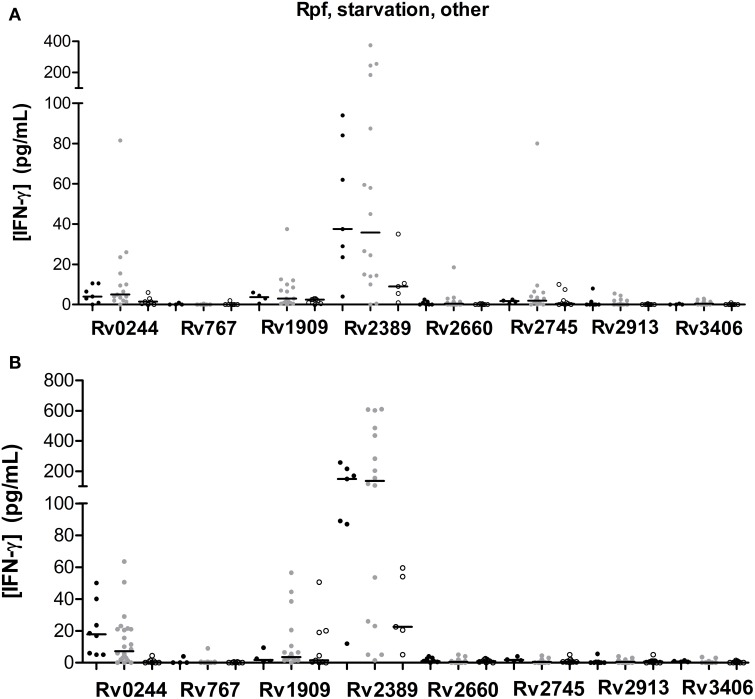
**Rpf, starvation and other stress response-associated antigen stimulated IFN-γ response (pg/ml) after short (A) or long-term (B) incubation of whole blood from individuals without LTBI (black dots), with LTBI (gray dots), and active TB patients (empty dots)**. The horizontal line represents the median.

We tested 1 Rpf, 1 starvation, and 6 other stress response-associated antigens (Figure 3). The response to some of those antigens did present statistical differences when compared among groups (Tables 3, 4). Rv2389, and Rv0244 in short and long-term estimulation, and Rv1909 in long-term stimulation induce high response in infected individuals. However, in some of them relevant production of IFN-γ after the stimulation in non-infected individuals was observed. Rv2389 accurately predicted 81% (13/16) of LTBI patients in long-term stimulation.

### Immunogenicity of IVE-TB antigens

**Figure 4:**
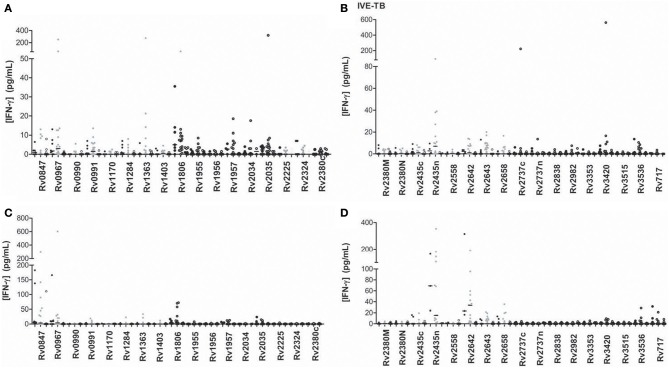
**IVE-TB antigen stimulated IFN-γ response (pg/ml) after short (A,B) or long-term (C,D) incubation of whole blood from individuals without LTBI (black dots), with LTBI (gray dots), and active TB patients (empty dots)**. The horizontal line represents the median.

We evaluated the immunogenicity of 34 IVE-TB antigens (Figure 4). The LTBI individuals were the group that most recognized IVE-TB antigens, although the response level was not very high (Tables 3, 4). In addition to the antigens that induce a significant IFN-γ response (Rv0967, Rv1806, and Rv1957 after short-term stimulation; and Rv0847, Rv2558, and Rv2642 after long-term stimulation), several antigens obtained IFN-γ response in infected individuals: Rv0847, Rv0990, Rv0991, Rv1363, Rv1955, Rv2034, Rv2035, Rv2435n, Rv2642, Rv2643, Rv2658, Rv3420, and Rv3536 after short-term stimulation; and Rv1363, Rv1806, Rv2435n, Rv2643, Rv25658, and Rv3536 after long-term stimulation. However, in some of them the amount of IFN-γ was not very high, and the regions of response overlapped with the response obtained in non-infected individuals.

### Comparison between QFN-positive and QFN-negative LTBI individuals

Given that the lack of specificity of the TST, we evaluated if the response of LTBI individuals to the 60 novel latency-related mycobacterial antigens varied according to the result of their QFN. There were four antigens to which the response was statistically different depending on the QFN result (Table 5) when used in short-term stimulation. Latently infected individuals responded to Rv2389 (*p* = 0.029) and Rv2435n (*p* = 0.050) in higher amounts when the individual presented a positive QFN, thus indicating to be promising LTBI biomarkers. However, the response to Rv2435n overlapped between the two groups. The other two antigens, Rv2660 and Rv2380M, induced a significant response in individuals with a negative QFN (*p* = 0.046 and *p* = 0.030, respectively), indicating to be possible candidates for remote infection. The response to Rv2380M barely overlapped in the two groups, whereas the response to Rv2660 clearly overlapped. Some of these antigens could be good candidates to be used in combination with the QFN.

**Table 5:** **Median levels of IFN-γ (pg/ml), minimum, and maximum values (in brackets) elicited in latently infected individuals depending on the QFN result**.

	**Positive QFN**		**Negative QFN**		
**Antigen**	***n***	**Median**	***n***	**Median**	***p*-value**
Rv2389	13	9.0 (0.0, 95.5)	5	0.5 (0.0, 2.5)	0.029
Rv2660	15	0.0 (0.0, 18.5)	6	1.0 (0.0, 3.5)	0.046
Rv2380M	13	0.0 (0.0, 1.0)	6	3.3 (1.0, 4.5)	0.003
Rv2435n	10	10.5 (0.0, 92.5)	7	1.0 (0.0, 39.0)	0.05

*N indicates the number of subjects in each group. Data were analyzed by Mann Whitney test for the comparison of patients with positive QFN and negative QFN. Only significant differences (p < 0.05) were included*.

### Effect of BCG vaccination in the response to studied antigens in individuals with and without LTBI

Among non-infected individuals, there were five antigens in which the response was different according to the vaccination status. Specifically, Rv717, Rv0570n, Rv2658, and Rv2643 induced a significant response in BCG vaccinated individuals (*p* = 0.043, *p* = 0.009, *p* = 0.041, *p* = 0.041, respectively). In contrast, it was the non-BCG vaccinated individuals who most responded to Rv2627 (*p* = 0.019). Interestingly, while the higher response to antigens in BCG vaccinated individuals was produced after short-term stimulation, the higher response elicited by non-vaccinated individuals was only produced after long-term stimulation.

Regarding the LTBI subjects, BCG vaccinated individuals elicited a significant response to the following antigens: Rv2035, Rv1471, Rv1957, and Rv2435n (*p* = 0.016, *p* = 0.020, *p* = 0.020, *p* = 0.015, respectively) when compared to non-BCG subjects. In all four antigens the significance was after overnight stimulation.

## Discussion

In this study we evaluated whether latency antigens induced a response which varied according to the group of individuals. Each category of antigens, that is, DosR regulon-encoded, TB reactivation-associated, Rpf, starvation, IVE-TB antigens and other stress response-associated TB antigens, contained at least one antigen whose statistical analysis was significative.

Regarding the control antigens we studied, in general, the response they induced was higher after 7 days of incubation. PPD and ESAT-6/CFP-10 were the antigens which induced a highest response, followed by the TB10.4 and finally Ag85A, which is in concordance with what Kassa et al. (2012) found. According to our finding, Chegou et al. (2012) observed that the majority of response to *M.tb* infection is largely driven by ESAT6/CFP-10, not by the other antigens that they used as controls (TB7.7, Ag85A/B, and HSP65), where the recognition was poor. Generally speaking, it was observed that infected individuals provided higher responses than those with the disease. In a study performed by Sutherland et al. (2013) it was found, instead, that PPD and ESAT-6/CFP-10 generated dominant responses but very few differences between active TB and LTBI subjects. In the present study, some antigens induced a lower response in active TB patients when compared with non-TB infected subjects. This fact could be explained by the criteria selection followed for including individuals in the non-infected group. In our study, patients from LTBI screening with negative QFN, but TST results under 10 mm (non BCG-vaccinated) or 15 mm (BCG-vaccinated) were considered non-infected, but in some cases some cross-reactivity with the antigens used as a control (including ESAT-6/CFP-10) and the latency antigens could not be rejected. Indeed, in general, in the group of non-infected individuals the IFN-γ responses against ESAT-6/CFP-10 are lower in patients with TST under 5 mm, than in patients with TST over 5 mm (data not shown). On the other hand, results in the literature regarding IFN-γ responses to the these antigens in active TB patients are inconsistent. Possible differences may reside in variations in host genetic makeup, *M.tb* strains, study methodologies or the extent of TB progression, with diminished IFN-γ production during advanced disease (Weir et al., 2003; Jabado and Gros, 2005; Tsenova et al., 2007; Winek et al., 2008; Day et al., 2011).

Among all *M.tb* recombinant antigens we studied, there is one which stands up significantly: Rv1733, which is a probable conserved transmembrane protein and is part of the DosR regulon. As it can be observed in Figure 1, this antigen induces a differentiated response between non-infected subjects, infected individuals and TB patients. Specifically, infected individuals are the ones that generate a highest response, followed by the non-infected, and by the patients with the active disease at the end. Rv1733 immunogenity has been previously analyzed, and a genomic study from Zvi et al. (2008) describes it as an immunodominant T cell antigen. Moreover, many authors agree that there is significantly higher T cell response in LTBI as compared to TB patients (Vordemeier et al., 1991; Leyten et al., 2006; Black et al., 2009; Schuck et al., 2009; Commandeur et al., 2011; Sutherland et al., 2013), and Rv1733 is one of the DosR antigens that induces a highest response. Interestingly enough, this response pattern against Rv1733 is produced after the stimulation during 24 h, which makes it very appealing to be used for diagnostic purposes. In contrast with our finding, Riaño et al. (2012) observed that LTBI and TB patients did not react to Rv1733. In another study with TB patients, a high response to Rv1733 was also obtained (Kassa et al., 2012).

Even though Rv1733 turned out to be a very immunogenic antigen, Rv2389, which belongs to the Rpf family, induced a high response in most of the individuals as well. Kassa et al. (2012) described that the Rv2389 was able to induce a high IFN-γ response in active TB patients. In fact, the immunoresponse to Rpf may play a protective role against bacilli reactivation (Riaño et al., 2012). Rv2389 was able to differentiate between non-infected individuals, individuals with LTBI and active TB patients when incubated with whole blood for 7 days, even though some overlapping is present. As observed by Chegou et al. (2012), active TB patients response was much lower than in non-TB individuals. Commandeur et al. (2011), demonstrated Rv2389 specific T cell response in long-term *M.tb* nonprogressors to active TB patients. Riaño et al., (2012), observed higher levels of IFN-γ in the supernatant of stimulated cells from LTBI compared to active TB patients. Huang et al. (2013) demonstrated that LTBI infected through household contacts possessed higher IFN-γ production to Rv2389c than did the community exposed individuals. In addition, QFN-positive individuals responded in a higher level to Rv2389 when compared to QFN-negative individuals. The Rv2389 ability of discriminate between these two groups of patients have been confirmed by receiver operating characteristics curve (ROC) analysis (area under curve = 0.877). Altogether, these findings indicate that Rv2389 would be a good biomarker of LTBI.

Concerning the recently identified IVE-TB antigens, the only research group that has studied their immunogenicity *in vitro* found that some of them induced high levels of IFN-γ (Commandeur et al., 2013). In our study, some antigens showed a certain difference when comparing the three groups of individuals. While the median value was quite low in all groups, a great number of antigens such as Rv0967, Rv1363, Rv1957, Rv2034, Rv3420, or Rv2642 among others were able to induce outstanding IFN-γ responses in some individuals. In concordance with the study performed by Commandeur and coworkers, it was the active TB patients the group who showed a lowest response. They observed that the individuals who generated a highest response were those that did it with ESAT-6/CFP-10 as well. They also observed that controls not exposed to *M.tb* and individuals with positive TST and negative response to ESAT-6/CFP-10 did not respond to IVE-TB antigens, which shows that there is a specificity linked to *M.tb* exposure. In our study, although some non-TB infected individuals produced IFN-γ after being stimulated with IVE-TB antigens, individuals with LTBI also responded as well. BCG vaccine was ruled out as the response trigger in subjects without LTBI, as T cells from non-BCG vaccinated individuals generated a response as well. However, due the difficulty of totally rule out the infection in the no LTBI individuals, we cannot reject that, alternatively, those responder non-BCG vaccinated individuals were really *M.tb* infected; and that the responder BCG-vaccinated individuals were, in fact, responding to the shared BCG antigens.

Interestingly, the response to Rv2380M and Rv2660 was higher in individuals with a negative QFN than subjects with a positive QFN, indicating that both antigens could be possible biomarkers for remote infection. In the opposite way, Rv2435n induced a higher response in subjects with positive QFN. It may be, therefore, a possible biomarker for recent infection.

Activated lymphocytes and effector T cells that produce IFN-γ from *M.tb* antigens sensitized individuals, persist for a limited time in the circulation once the antigen is cleared (Pathan et al., 2001). It is thought that central memory T cells, but not effector ones, may take several days (rather than hours) to produce effector cytokines (Kaech et al., 2002; Dheda et al., 2007). This is because, the commercial IGRAs are thought to reflect more recent, rather than remote infections. Therefore, contrary to the findings of the TST, in cases of remote infection, the IFN-γ level did not increase during the short period of exposure to the antigen in the *ex vivo* IFN-γ assay at baseline. For these reasons we chose to stimulate short and long term the blood samples with the different latency-related antigens. Interestingly, the higher IFN-γ responses have been obtained after long-term stimulation instead of short-term stimulation: Rv1733, Rv3862, Rv2662, Rv0244, Rv2389, Rv1909, Rv2435n, Rv0847, Rv0967, Rv1806, and Rv2642.

As far as we know, only Goletti et al. (2010) assessed the comparison between individuals recently and remotely infected to five latency mycobacterial antigens. They found that Rv2628 was able to differentiate recent from remote infection, being the individuals with remote infection the group that showed significantly higher IFN-γ whole blood responses. In a very preliminary results, using well TB status characterized individuals, we have observed that responses to some antigens (Rv2380M, Rv0967, Rv2435n, and Rv2913) could differentiate between recent and remote infection (data not shown).

The fact of not obtaining response to an antigen that other studies identify as immunogenic, can be due to different host immune responses, *M.tb* strains and variations in the methodology used (Ottenhoff et al., 1998; Caws et al., 2008; Homolka et al., 2010), and also some factors such as ethnicity (host genetics), nutritional status, and microbial environment (Sutherland et al., 2013). The discordance in results between studies could be also attributed to the lack of gold standard for defining LTBI, and the consequent heterogenicity in the study population included in the different studies. The difficulty of establishing a group of LTBI is demonstrated by the criteria followed by the different authors: Leyten et al. (2006) included both patients from contact-tracing studies and also from screening studies; Chegou et al. (2012) included contact-tracing studies individuals, where neither TST nor QFN results were available; Commandeur et al. (2013) included TST positive patients, with exposure to *M.tb* and or with history of traveling to high TB incidence countries; and Sutherland et al. (2013) included household contacts of TB patients or by random community selection or from HIV care clinics with TST higher than 10 mm in HIV negative, and higher than 5 in HIV positive (independently of the BCG status). According to a recent study (Sutherland et al., 2013), which includes individuals from different sub-Saharan African countries, despite possible differences in the criteria of study subjects, there were variations between sites in regards to antigen reactivity, suggesting that need to be considered.

In order to ensure the validity of the promising antigens, we decided to study if some antigens induced a different response depending on whether the individual had been vaccinated with BCG or not. Among the antigens that distinguished between non-infected individuals, infected individuals and patients with TB disease, three of them were also identified when we analyze the effect of the BCG: Rv2627, Rv1471, and Rv1957. In order to measure the magnitude of the BCG influence, the response of non-vaccinated individuals was assessed. Being *p* > 0.05 and the charts showing an overlapping of the response between the three groups it seems that BCG has a considerable influence in the results (data not shown).

The effect of the BCG on the immune response against latency *M.tb* antigens has been studied by other authors. Lin et al. (2007) found that, although the homology between the DosR regulon from the BCG strain and from *M.tb* was very high, BCG-vaccinated individuals did not present immune response against DosR. Instead, individuals exposed to *M.tb* did respond to DosR. Thus, it seems that the response to antigens linked to the control of LTBI is only generated when there is an exposure to *M.tb*, and it does not depend on whether the individual has been immunized by the BCG, probably because BCG fails to establish long-lived latent infections, and therefore it may not express (or under express) these antigens *in vivo* following vaccination (Honaker et al., 2008). However, this issue warrants further investigation.

The current study presents certain limitations which are worth mentioning. In the first place, it is worth highlighting the difficulty found in the classification of the individuals according to their TB status, specially among BCG-vaccinated individuals, since there is no gold standard assay for LTBI diagnosis. We therefore cannot rule out in some cases a misclassification. Secondly, it seems that some of the studied antigens could present certain lack of specificity; they could be shared in BCG strain and also in other mycobacteria (Lin et al., 2009), since some non-infected and non BCG-vaccinated individuals responded. Anyway, it is not clear whether the cross-reactivity to latency antigens in *M.tb* naive people contributes to the natural protection developed in 90% of the individuals who are infected but do not progress to active TB (Fine, 1995; Brandt et al., 2002). Thirdly, the sample size we could include was certainly limited for some antigens, including some antigens found as promising. Another limitation of our work lies in the fact that we only evaluated the immunoresponse in terms of IFN-γ production by T cells. Combination of other cytokines with IFN-γ can strengthen the diagnostic potential of *M.tb* antigen (Goldsack and Kirman, 2007). However, despite these limitations, this work obtained strong conclusions identifying potential antigens as candidates for further validation studies.

In conclusion, after screening the potential antigenicity in subjects across the spectrum of TB, we could identify promising antigens in all groups of antigens studied. Rv1733, which is encoded in DosR regulon, turned out to be very immunogenic and able to discriminate between the three defined TB status, thus considered a candidate biomarker. Rv2389 and Rv2435n, belonging to Rpf family and IVE-TB group of antigens, respectively, also stood out as LTBI biomarkers. Further work needs to be done in order to support our hypothesis and to have a pattern of host responses available so that by testing the response to a set of *M.tb* antigens we can define the TB status and make a clinical decision.

## Author contributions

All authors listed contributed substantially in the conception or design of the work (Tom H. M. Ottenhoff, José Domínguez) or the adquisition of data (Irene Latorre, Kees L. C. M. Franken, Jéssica Díaz, Maria Luiza de Souza-Galvão, Irma Casas, José Maldonado, Cèlia Milà, Jordi Solsona, M. Ángeles Jimenez-Fuentes, Neus Altet, Alícia Lacoma, Juan Ruiz-Manzano, Cristina Prat), analysis (Vicente Ausina, M^a^del Mar Serra-Vidal, José Domínguez, Tom H. M. Ottenhoff), or interpretation of data (M^a^del Mar Serra-Vidal, José Domínguez, Tom H. M. Ottenhoff); in drafting the work or revising it critically for important intellectual content; in doing final approval of the version to be published; and in agreement to be accountable for all aspects of the work in ensuring that questions related to the accuracy or integrity of any part of the work are appropriately investigated and resolved.

### Conflict of interest statement

Tom H. M. Ottenhoff is coinventor of a *M.tb* latency antigen patent, which is owned by Leiden University Medical Center, but receives no financial benefits from this. The authors declare that the research was conducted in the absence of any commercial or financial relationships that could be construed as a potential conflict of interest.
